# Molecular changes during progression from nonmuscle invasive to advanced urothelial carcinoma

**DOI:** 10.1002/ijc.32737

**Published:** 2019-11-14

**Authors:** Gottfrid Sjödahl, Pontus Eriksson, Oliver Patschan, Nour‐Al‐Dain Marzouka, Lovisa Jakobsson, Carina Bernardo, Kristina Lövgren, Gunilla Chebil, Ellen Zwarthoff, Fredrik Liedberg, Mattias Höglund

**Affiliations:** ^1^ Department of Translational Medicine, Division of Urological Research Lund University Lund Sweden; ^2^ Department of Urology Skåne University Hospital Skåne Sweden; ^3^ Division of Oncology and Pathology, Clinical Sciences Lund University Lund Sweden; ^4^ Department of Pathology Erasmus MC Cancer Institute, Erasmus Medical Center Rotterdam The Netherlands

**Keywords:** bladder cancer, urothelial carcinoma, molecular subtypes, progression, mutation

## Abstract

Molecular changes occurring during invasion and clinical progression of cancer are difficult to study longitudinally in patient‐derived material. A unique feature of urothelial bladder cancer (UBC) is that patients frequently develop multiple nonmuscle invasive tumors, some of which may eventually progress to invade the muscle of the bladder wall. Here, we use a cohort of 73 patients that experienced a total of 357 UBC diagnoses to study the stability or change in detected molecular alterations during cancer progression. The tumors were subtyped by gene expression profiling and analyzed for hotspot mutations in *FGFR3*, *PIK3CA* and *TERT*, the most frequent early driver mutations in this tumor type. *TP53* alterations, frequent in advanced UBC, were inferred from p53 staining pattern, and potential genomic alterations were inferred by gene expression patterns at regions harboring frequent copy number alterations. We show that early driver mutations were largely preserved in UBC recurrences. Changes in *FGFR3*, *PIK3CA* or *TERT* mutation status were not linked to changes in molecular subtype and aggressive behavior. Instead, changes into a more aggressive molecular subtype seem to be associated with p53 alterations. We analyze changes in gene expression from primary tumors, to recurrences and progression tumors, and identify two modes of progression: Patients for whom progression is preceded by or coincides with a radical subtype shift, and patients who progress without any systematic molecular changes. For the latter group of patients, progression may be either stochastic or depending on factors already present at primary tumor initiation.

## Abstract 2

**What's new?**

Molecular changes occurring during invasion and clinical progression of cancer are difficult to study longitudinally in patient‐derived material. A unique feature of urothelial bladder cancer is that patients frequently develop multiple nonmuscle invasive tumors, some of which may eventually progress to invade the muscle of the bladder wall. Here, the authors perform multi‐level longitudinal analyses on patients with progression from non‐muscle invasive to advanced disease and describe novel modes of progression related to shifts in molecular profiles. Combined with the theory of field cancerization, these results identify limitations in predicting clinical progression based on molecular data from non‐muscle invasive tumors.

**Abbreviations**
Ba/Sq: basal/squamous
BCG: Bacillus Calmette‐Guerin
CIS: carcinoma *in situ*
DEG: differentially expressed gene
FFPE: formalin‐fixed paraffin‐embedded
GU: genomically unstable
IHC: immunohistochemistry
Mes‐like: mesenchymal‐like
MIBC: muscle‐invasive bladder cancer
NMIBC: nonmuscle invasive bladder cancer
RC: radical cystectomy
SAM: significance analysis of microarrays
Sc/NE: small‐cell/neuroendocrine‐like
TMA: tissue microarray
TUR‐BT: trans‐urethral resection of bladder tumor
UBC: urothelial bladder cancer
Uro: urothelial‐like

## Introduction

Surface epithelia are composed of clonal areas that replenish the tissue during normal homeostasis.^1^, ^2^, ^3^ Recurrent *in situ* and papillary lesions occurring in these tissues within the same patient are either independent, originating from different clonal units or may have a shared clonal origin. If the site of tumor recurrence is different from that of the primary tumor, this is a manifestation of field cancerization,^4^ either due to single clone expansion or due to independent fields, that is, oligo‐clonal acquisition of tumor‐initiating capacity.^5^ Studies on the genetic heterogeneity of the premalignant field in patients with urothelial bladder cancer (UBC) have given important insights into the processes behind tumor initiation and favor a clonal origin.^6^, ^7^, ^8^, ^9^ The shared clonal origin of nonmuscle invasive bladder cancer (NMIBC) recurrences has been repeatedly demonstrated^10^, ^11^, ^12^ even though tumors usually occur in different locations within the bladder.^13^ Taken together, a model is supported in which tumors develop semi‐independently out of a premalignant field that is usually clonal in nature but has additional heterogeneity that manifests as genetic differences between recurrences.^14^, ^15^ Thus, NMIBC is an ideal system to study the stability of molecular characteristics in multiple local recurrences originating from the same premalignant epithelium. In clinical management, the least aggressive forms of NMIBC (Ta, low‐grade) are lacking invasive potential even without resection.^16^ On the other hand recurrences frequently occur, also in patients with low‐grade tumors, many experiencing over 10 recurrences over the course of many years.^17^, ^18^ The risk of progression to muscle‐invasive bladder cancer (MIBC) and cancer‐specific death for these patients is minimal but increases to 18% when including stage T1 high‐grade disease.^19^ High‐risk NMIBC patients are treated with intravesical Bacillus Calmette‐Guerin (BCG) or even radical cystectomy (RC). Predictors of increased risk include pathological stage T1, high pathological grade,^20^ molecular risk scores^21^, ^22^ and an aggressive, Genomically Unstable or Basal/Squamous, molecular subtype.^23^ Recurrent as well as progressive disease has also been studied longitudinally in clinical^17^, ^24^ and molecular studies.^11^, ^18^, ^25^, ^26^ However, the proportion of patients that progress in these existing studies is low. Therefore, they do not fully capture the variability between the patients and tumors that eventually progress. This knowledge gap poses a problem when data from one single index tumor is selected as a basis for risk prediction at the patient‐level.

The aim of our study was to use recurrent NMIBC as a model system to study the longitudinal stability or change in molecular subtypes and driver mutations of recurrent tumors arising in the same epithelial tissue. We correlate the findings with the patient's clinical history, the timing of recurrences and intravesical treatment courses. We add a new perspective compared to previous studies^11^, ^18^, ^25^, ^26^ by studying exclusively the population that eventually progress to advanced disease.

## Materials and Methods

### Patient and tumor characteristics

**Figure 1:**
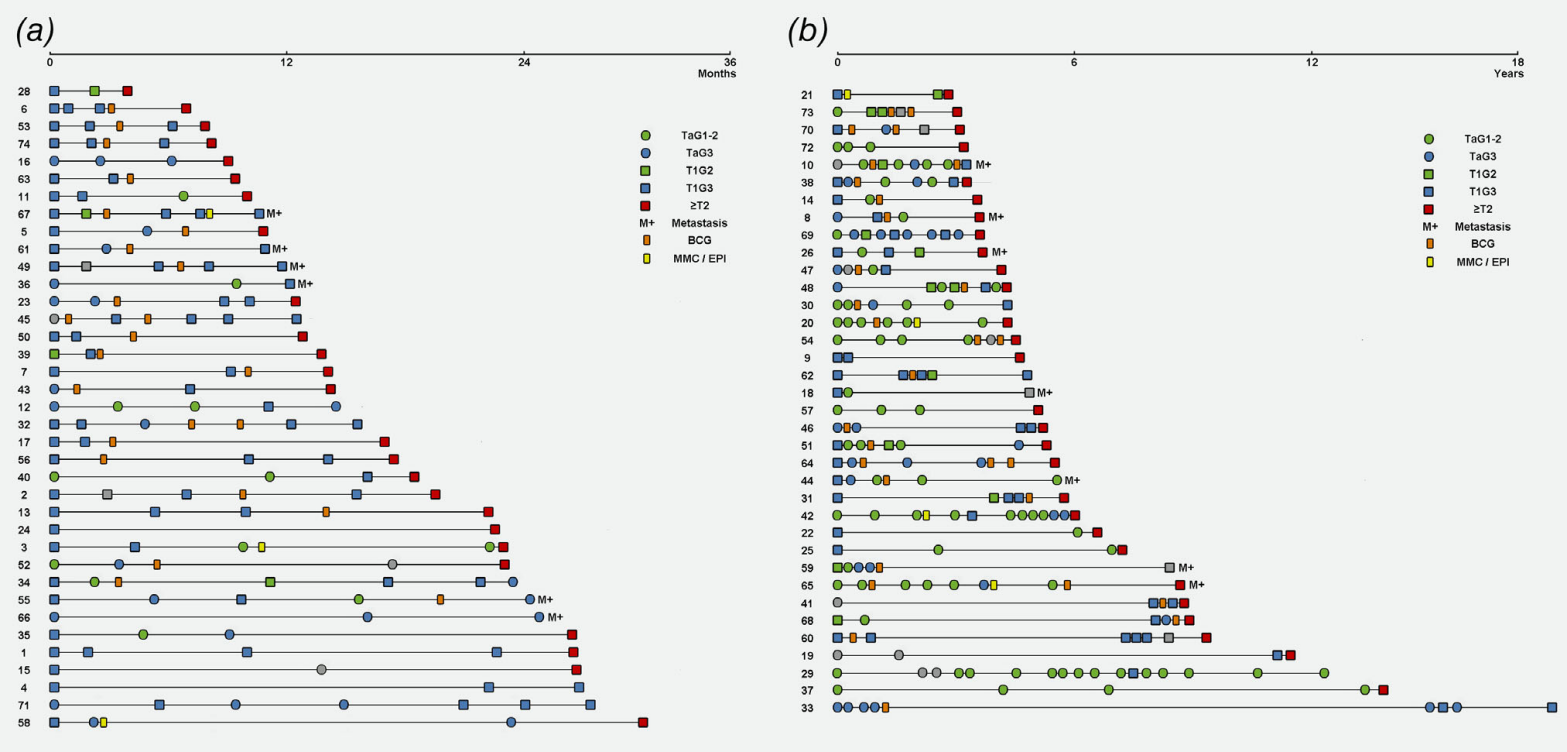
Chronologic disease course for 73 patients with progressive UBC. Patient timelines are shown divided by median time to progression into (*a*) early progressors with less than 3 years between primary tumor and progression, and (*b*) late progressors, with more than 3 years to progression. Squares and circles represent tumor manifestations, whereas rectangles represent intravesical treatment courses. Placement of symbols that would overlap due to short time between events has been adjusted minimally. [Color figure can be viewed at http://wileyonlinelibrary.com]

We identified 73 patients treated for a primary NMIBC between 1989 and 2013 in seven hospitals in the Southern Health Care region in Sweden that subsequently developed NMIBC recurrences, and finally progressed. Progression was defined as a diagnosis of MIBC, metastasis (M+) or that the patient underwent RC. Our study thus adheres to established definitions,^27^ except that we did not consider an increase in tumor stage from Ta/CIS to T1 or an increase in grade to constitute a progression event. The study was approved by the local ethical review board. Pathological review was performed by an experienced uropathologist (GC) using the WHO 1999 grading system. The 73 patients presented with 357 tumors, with a minimum of two, a maximum of 17 and a median of four tumors per patient. The majority (45/73, 62%) of the patients received at least one full induction course of intravesical BCG, and a minority (7/73, 10%) received intravesical chemotherapy regimens (full courses of Mitomycin‐C or Epirubicin). The advent of BCG in favor of instillations with chemotherapy and the introduction of re‐resection in TaG3/T1‐disease were successively applied according to hospital routines, until the first national bladder cancer guidelines were published in 2013. Data and timing of tumor stage, tumor grade, intravesical treatment and type of progression are summarized in Figures 1 *a* and 1 *b*. Clinical data are available in Supporting Information Table S1.

### Immunohistochemistry

From the 357 tumors, 300 formalin‐fixed paraffin‐embedded (FFPE) transurethral resection of bladder tumor (TUR‐BT) tissue blocks were obtained of which 271 contained sufficient tissue for immunohistochemistry (IHC)‐based molecular classification. For each TUR‐BT, one tissue block from a single tumor was used for all molecular analyses. Tumors were embedded in tissue microarrays (TMAs) with two 1.0 mm cores per tumor, although for 46 tumors only a single TMA core was available. Hematoxylin and Eosin staining, and IHC against 16 proteins were applied (CCNB1, CNND1, CDH1, EPCAM, ERBB2 FGFR3, FOXA1, GATA3, KRT14, KRT5, p16, RB1, p53, TUBB2B, VIM and ZEB2; Supporting Information Table S2). IHC‐based molecular subtype classification was performed by calculating 4‐marker subtype scores as described previously,^28^ using prespecified cut‐offs for class assignment. Briefly, basal/squamous‐like (Ba/Sq) subtype was assigned to tumors with high KRT5, KRT14 and low FOXA1, GATA3. Mesenchymal‐like (Mes‐like) subtype was assigned to tumors with high VIM, ZEB2 and low CDH1, EPCAM. Small‐cell/neuroendocrine‐like (Sc/NE) subtype was assigned to tumors with high EPCAM, TUBB2B and low CDH1, GATA3. Tumors not assigned to either of these three subtypes are “Luminal‐like” and were further classified as Urothelial‐like (Uro) if CCND1, FGFR3 and RB1 showed strong and p16 weak staining, or genomically unstable (GU) if they showed the opposite staining intensities. Thus, the five subtype scores represent condensed molecular data points, each contributing information essential for assessing the tumor phenotype. We considered patients to have stable subtypes if each tumor had an internal correlation of subtype scores >0.75 compared to the mean subtype scores of the patient. The cut‐off was applied as a quality control to ensure that subsequent analyses were based on robust subtype shifts and not merely nominal changes due to a threshold effect in classification. The cut‐point was selected empirically, prior to analyses, and is data set specific. IHC data were manually inspected to ensure that substantial subtype shifts were not excluded in this process. All IHC data are available in Supporting Information Table S1.

### Transcriptomics and RNA‐based molecular subtype classification

Tumor areas were macrodissected from FFPE sections, RNA was extracted and 200 tumors were analyzed using Affymetrix Gene ST 1.0 microarrays. Macrodissection and extraction protocols were as described previously.^28^ Samples with a total RNA yield of 0.5 μg or more were amplified, labeled (SensationPlus kit, Thermo Fisher, Waltham, MA) and hybridized to Gene ST 1.0 arrays (Thermo Fisher). The data set was preprocessed by RMA‐normalization followed by COMBAT adjustment of labeling batches, and median centered for heatmap visualization. Molecular subtype classification was performed into the Lund Taxonomy subtypes described previously,^29^ by classifying single‐samples into the main molecular subtypes Uro, GU, Ba/Sq, Mes‐like and Sc/NE and the subclasses UroA, UroB and UroC, for samples first classified as Uro. The consensus molecular subtypes of MIBC were also applied.^30^ Molecular classification results are available in Supporting Information Table S1.

### Mutation analysis

We used DNA from FFPE extractions of 220 tumors to perform a battery of 15 snapshot assays to detect the nine most frequent mutations in *FGFR3*, the three most frequent mutations in *PIK3CA*, and the three most frequent mutations in the *TERT* promoter. The assays have been described and validated previously,^31^, ^32^, ^33^ and mutation calling based on chromatogram peaks was done blinded to the identity of the samples. DNA extraction yield ranged from 0.1 to 17.1 μg, and showed weak association with the number of detected mutations (Supporting Information Table S3). Only tumors with an unambiguous peak indicating the clear presence of a mutant allele were annotated as carrying the mutation. We identified 11 low or ambiguous peaks that were considered wild‐type in all analyses. Mutation data are available in Supporting Information Table S1. To evaluate TP53 aberrations we used IHC staining with the DO7 antibody (Dako, Carpinteria, CA). Expression patterns were evaluated as wild type, overexpression, complete absence or cytoplasmic, as previously described.^34^ The nonwild type categories were grouped to indicate p53 altered cases.

### Statistical analyses

Statistical tests were performed using R, and statistical tests of significance were two‐sided at an α = 0.05 unless otherwise specified. Fisher's exact test was used to test differences in proportions of categorical data. Differentially expressed genes were identified using paired *t*‐test in Multi Experiment Viewer (MeV), with genes showing *q* < 0.01 (using 1,000 permutations) considered as significant.

## Results

### Clinical course of progression

We studied the clinical course of recurring UBC by chronologically ordering the timelines of 73 patients, divided by median time to progression into early and late progressors (Figs. 1 *a* and 1*b*), and indicating tumor stage, tumor grade and intravesical treatment. Organizing the data in this manner showed that patients progressing to advanced disease within approximately 3 years were enriched for primary tumors that had invaded across the basal membrane (Stage T1), whereas patients with later progression more frequently had primary tumors confined to the urothelium (Stage Ta, *p* = 0.03). It also became evident that patients with multiple low grade (G1‐G2) recurrences tended to fall into the late progression group (Fig. 1 *b*). Only three patients in the early progression group experienced more than one G1–G2 tumor before progression, while 19 patients in the late progression group did (*p* = 3.2 × 10^−5^). Although the patients with early progression had on average fewer NMIBC recurrences (2.0 *vs*. 3.6), their rate of recurrences was more than double that of the late progression group (1.4 *vs*. 0.6 recurrences per year). We observed no difference in the type of progression (MIBC, M+ or RC) or the use of intravesical treatment in the early *vs*. late progression groups.

### Changes in molecular subtype classification occur in a subset of patients that progress

**Figure 2:**
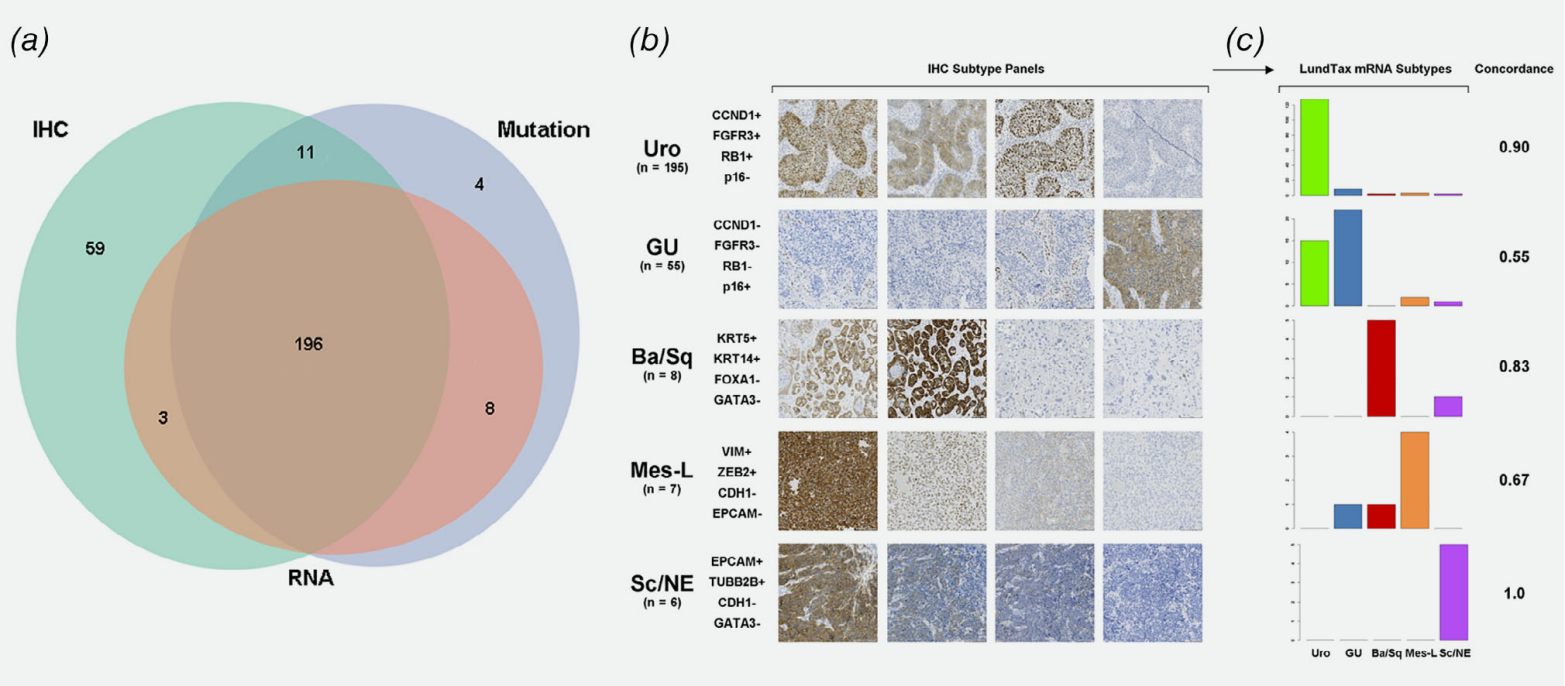
Overlap of data types and concordance between IHC‐ and mRNA‐based molecular subtype classification. Venn diagram in (*a*) counting tumors for which IHC (green), RNA (blue) and mutation (red) data are available. All three data types overlapped for 193 tumors. (*b*) The numbers of tumors classified by IHC into each of the five major UBC molecular subtypes are shown. The four IHC stains used for subtyping are shown for a typical case of each type. The tumor used to exemplify the Urothelial‐like subtype in panel b is also shown in Supporting Information Figure S3. Barplots in (*c*) show the proportion of LundTax mRNA subtypes within the IHC subtypes, and the per‐subtype concordance between the two classification methods. [Color figure can be viewed at http://wileyonlinelibrary.com]

Multiple immunostaining, transcriptomic and mutation data from every tumor for which we could obtain material of sufficient quality and amount showed a large overlap such that 196 tumors were covered by all three data types (Fig. 2 *a*). Immunohistochemistry (IHC)‐based classification of molecular subtypes (Fig. 2 *b*) achieved a high concordance with respect to mRNA‐based subtypes (0.82, 164 of 200 cases with concordant classification; Fig. 2 *c*). Discordant classification was mainly (23/36) observed between the two luminal‐like subtypes urothelial‐like (Uro) and genomically unstable (GU), with 15 of the tumors classified as GU by IHC and Uro by mRNA, and 8 tumors classified as Uro by IHC and GU by mRNA. This is in accordance with previous findings in which UroC, a subset of Uro tumors, has been shown to be difficult to discriminate from GU.^28^

**Figure 3:**
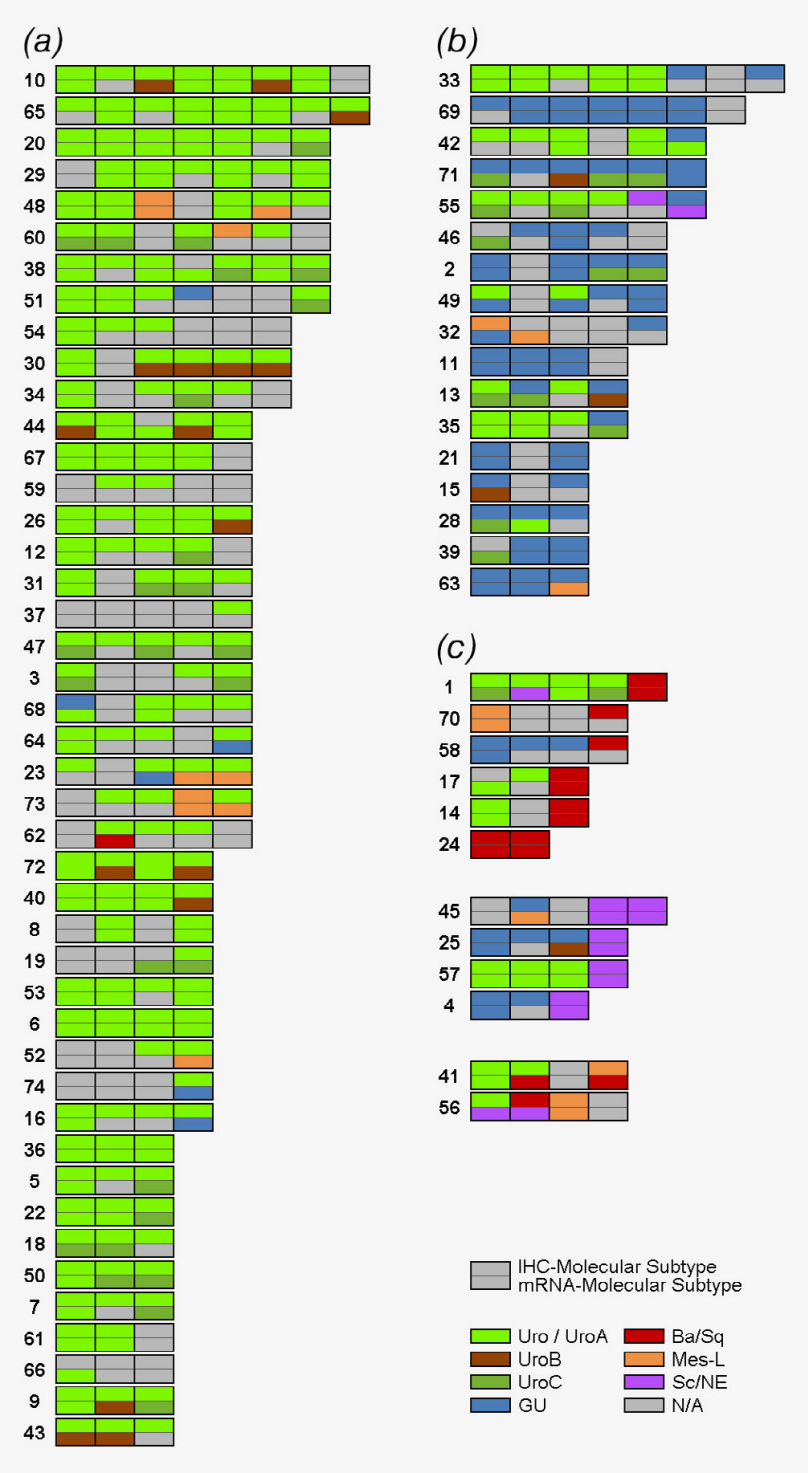
Changes in molecular subtype classification in primary, recurrent and progressive bladder tumors. Each row represents one patient and each split box represents a tumor: The upper half colored by the IHC‐based molecular subtype and the lower half colored by the mRNA‐based subtype. The data is organized into (*a*) patients for whom the last available tumor is classified as Urothelial‐like (Uro) by IHC, (*b*) patients for whom the last available tumor is classified as GU by IHC and (*c*) patients for whom the last available tumor is classified as basal/squamous‐like (Ba/Sq), mesenchymal‐like (Mes‐like) or small‐cell/neuroendocrine‐like (Sc/NE) by IHC. [Color figure can be viewed at http://wileyonlinelibrary.com]

We ordered patients by the molecular subtype of the last classified tumor and investigated to what extent classification changed over the disease course (Fig. 3). Patients for whom the last classified tumor was of the Uro subtype tended to have more prior tumors, almost exclusively also classified as Uro (Fig. 3 *a*). Since the Uro subtype contains most cases with low stage (Ta) and grade (G1–G2) it is logical that these patients experience more tumors before progression. In these patients, only two recurrences were classified into a different subtype by both IHC‐ and mRNA‐based subtyping, that is, very few definite subtype shifts were observed. Patients for whom the last classified tumor was of the GU subtype tended to have prior tumors of either the GU or the Uro subtypes (Fig. 3 *b*). Patients for whom the last classified tumor was of the Basal/Squamous‐like (Ba/Sq), Mesenchymal‐like (Mes‐like) or Small‐cell/Neuroendocrine‐like (Sc/NE) subtypes, had few prior tumors that could be of any subtype (Fig. 3 *c*).

Although molecular subtype classification was stable within most patients, the assignment of one subtype to each tumor may result in different subtype labels for tumors simply because they are close to the threshold between two subtypes. To avoid false interpretations of such threshold effects as subtype change, we quantified phenotypic differences by analyzing quantitative scores from the IHC‐, and mRNA‐classifiers. For each tumor, we compared the values on the five subtype scores to the mean subtype scores from the same patient. This value was used as a similarity measure in the subtype classification space. As shown in Supporting Information Figure S1 this measure identifies a major group of patients for which all the tumors were internally highly similar and a minor group of patients in which one or several outlier tumors deviated from the rest. We consider only the subtype changes in the latter patient category to be valid, whereas the nominally different subtype classifications in the stable group were disregarded as threshold effects. Thus, in the full cohort, 20 patients (27%) had at least one subtypes change before progression that was validated with this approach. For seven patients, we identified valid subtype shifts both on the IHC and mRNA level, whereas five and eight patients showed validated shifts only at the IHC‐, and mRNA levels, respectively.

### Stability of driver mutations over multiple recurrences is independent of molecular subtype

**Figure 4:**
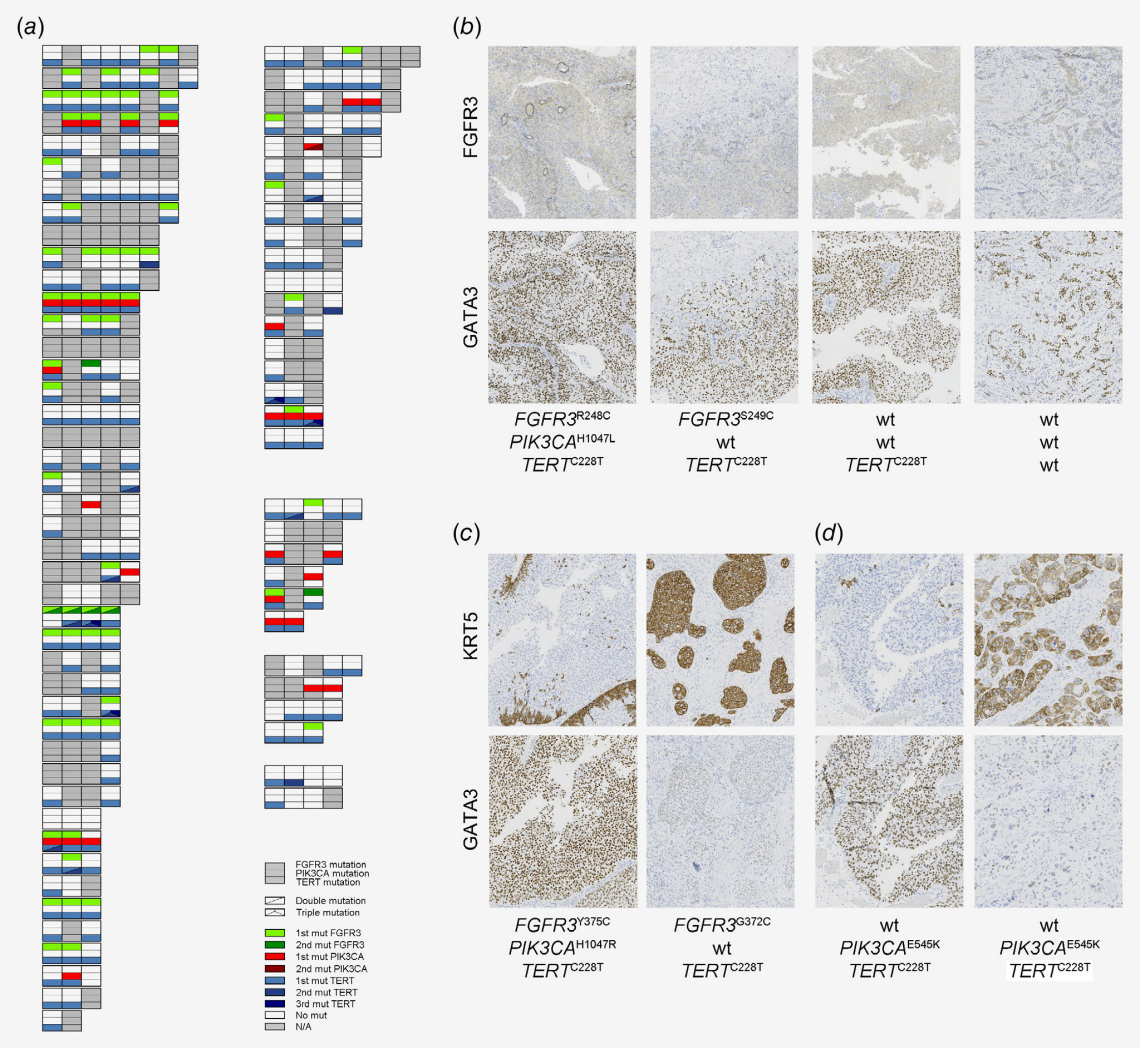
Mutations in recurrences are independent of molecular subtype stability. Each row in (*a*) represents a patient and each split box represents a tumor: the upper third is colored by *FGFR3* mutation status, the middle third by the *PIK3CA* mutation status and the lower third by the *TERT* mutation status. Patients and tumors are organized as in Figure 3. (*b*) Molecular data for a patient with four tumors stably classified as Urothelial‐like (UroA–UroA–UroA–UroB), for which every tumor's mutation status differs from the rest. (*c*) Molecular data for a patient with a primary UroA tumor that changed subtype to basal/squamous in the progression tumor. The two tumors showed different *FGFR3* and *PIK3CA* mutations. (*d*) Molecular data for a patient with a primary GU tumor that changed subtype to Basal/Squamous in the progression tumor. The two tumors showed identical mutation profile. [Color figure can be viewed at http://wileyonlinelibrary.com]

Next, we analyzed hotspot driver mutations in *FGFR3*, *PIK3CA* and *TERT* in 220 tumors and detected 70 *FGFR3* mutations, 32 *PIK3CA* mutations and 185 *TERT* mutations (Supporting Information Table S4). Mutations were not enriched in primary, recurrent, or progression tumors (Supporting Information Fig. S2), but the frequency of *FGFR3* mutations was, as expected, significantly elevated in tumors classified as Uro (40%), and lowest (7%) in tumors classified as GU (Supporting Information Fig. S2B). Mutations in *PIK3CA* were more frequent in tumors classified as Ba/Sq (57% *vs*. 13% in non‐Ba/Sq) although this proportion was based on only four of seven Ba/Sq tumors carrying *PIK3CA* mutations. Mutations in *TERT* were not associated with subtype (Supporting Information Fig. S2B). Tumor purity scores, calculated using the ESTIMATE tool,^35^ were associated with *FGFR3*, but not *PIK3CA* or *TERT* mutations, which could be explained by a positive association between the Uro subtype and tumor purity (Supporting Information Table S3). In longitudinal analyses, a third of the patients with mutation data from more than one tumor (23/65, 35%) had identical mutation status in all tumors (Fig. 4 *a*). An additional 25% (16/65) differed by a single mutation compared to the other tumors from the same patient. Longitudinal analyses also revealed that changes in molecular subtype were not associated with changes in mutation status of these genes. Of the tumors that changed molecular subtype and had available mutation data, 52% (12/23) had identical mutation status to the last prior tumor, 39% (9/23) differed in the status of a single mutation and only two of 23 (9%) differed by more than one mutation (Supporting Information Table S5). To exemplify the various observed scenarios, Figure 4 *b* shows one patient with four phenotypically stable Uro tumors with different mutation status in every tumor. A second patient with a change in subtype from Uro to Ba/Sq, demonstrated different mutation status in both *FGFR3* and *PIK3CA* (Fig. 4 *c*). A third patient with a change in subtype from GU to Ba/Sq, had identical mutation status of all three genes (Fig. 4 *d*). Taken together, most patients showed similar mutations in recurrent tumors but a minor group showed highly variable mutations. These mutation patterns did not overlap with phenotypic changes suggesting that they are independent.

### BCG treatment and changes in molecular subtype classification

Therapy changes the conditions for tumor growth and may induce change either directly or through selection. Of the patients with a robust subtype change, 13 received BCG while 5 did not. This proportion did not differ from the patients without any subtype shifts, of which 32 received BCG and 21 did not (*p* = 0.41). To test the association between subtype change and BCG at the tumor level, we counted all sequential instances of tumors with subtype classification data, that is, every instance where both a subtype change and BCG treatment could have occurred. We then classified those instances based on if a subtype change occurred, if BCG treatment occurred, or both. We observed that 38% (11/29) of such instances with subtype change coincided with BCG, compared to 21% (37/174) of instances without subtype change (*p* = 0.060). Supporting Information Table S5 lists the subtype changes and changes in mutation status according to BCG treatment and progression type (RC, MIBC or M+). To account for the fact that many patients either did not experience any subtype shifts, or did not receive any BCG, we repeated the tumor level analysis only in the 18 patients that experienced a subtype change (11/29 coinciding *vs*. 4/27 not coinciding, *p* = 0.072), and also in the 13 patients who both experienced subtype change and received BCG (11/20 coinciding *vs*. 4/18 not coinciding, *p* = 0.052). Thus, we could not find any statistically robust association between BCG treatment and shifts in molecular subtype.

### p53 staining is consistent in recurrences but may be abnormal upon shifts to aggressive subtypes

While *FGFR3*, *PIK3CA* and *TERT* are frequent drivers in early‐stage UBC (NMIBC), the most commonly mutated gene in MIBC is *TP53*. The D07 p53 antibody assay that detects TP53 alterations was applied with 97% concordance between TMA‐core pairs (Supporting Information Table S6). Abnormal p53‐pattern was observed in 62 of 277 tumors (22%) with highly significant enrichment in the GU subtype compared to Uro (Supporting Information Fig. S2). Within Uro, p53‐altered pattern was also significantly enriched in the UroC subset compared to UroA/B (*p* = 9 × 10^−4^). Longitudinally, 61% (45/69) of patients with data from multiple tumors showed wild‐type p53‐pattern in all tumors, whereas 10 patients (14%) showed p53‐abnormal pattern in all tumors, and 17 patients (25%) had both wild‐type and altered tumors. A resampling test revealed that in this dataset p53‐altered tumors were clustered among few patients to a higher degree than what would be expected by chance (*p* < 10^−5^). In line with this stability, there was no significant overall difference in frequency of abnormal patterns for progression tumors (15/41, 37%), compared to primary tumors (15/43, 35%) or recurrences (31/130, 24%). The likelihood of p53‐altered pattern was also significantly increased if the previous tumor was p53‐altered compared to what would be expected without knowledge of prior p53‐status (odds ratio = 6.9, 95% CI: 2.8–16.9). At the patient level, there was a trend toward positive association between the occurrence of any change in molecular subtype and any change in p53‐status (*p* = 0.059). We tested the association between subtype change and p53‐status at the tumor level by comparing all sequential tumors with both data types, that is, every instance where both a subtype change and change in p53‐status could have occurred. We then classified those instances based on if a subtype change occurred, if a change in p53‐status occurred, or both. This resulted in nine instances of subtype change coinciding with a change in p53‐status—a significantly higher number than expected in the absence of an association (*p* = 0.0016). In Supporting Information Figure S3, we highlight one such patient. This patient had the longest time to progression in the cohort due to over 10 years of remission after BCG. The patient's first four tumors were *FGFR3* ^wt^ Ta tumors of the Uro subtype. The first relapse after BCG, tumor number five, had acquired *FGFR3* ^R248C^ mutation, still within the Uro phenotype. Tumors number six and eight were of stage T1 instead of Ta, had p53 overexpression, and a molecular subtype shift from Uro to GU. This case suggests heterogeneous origins of different tumors representing one *FGFR3* ^wt^, one *FGFR3* ^R248C^ and one *FGFR3* ^wt^/*TP53*‐altered genomic state, correlating with the transition from early Ta‐Uro tumors to post relapse T1‐GU tumors.

### Recurrences show coordinated gene expression at loci harboring copy number alterations

**Figure 5:**
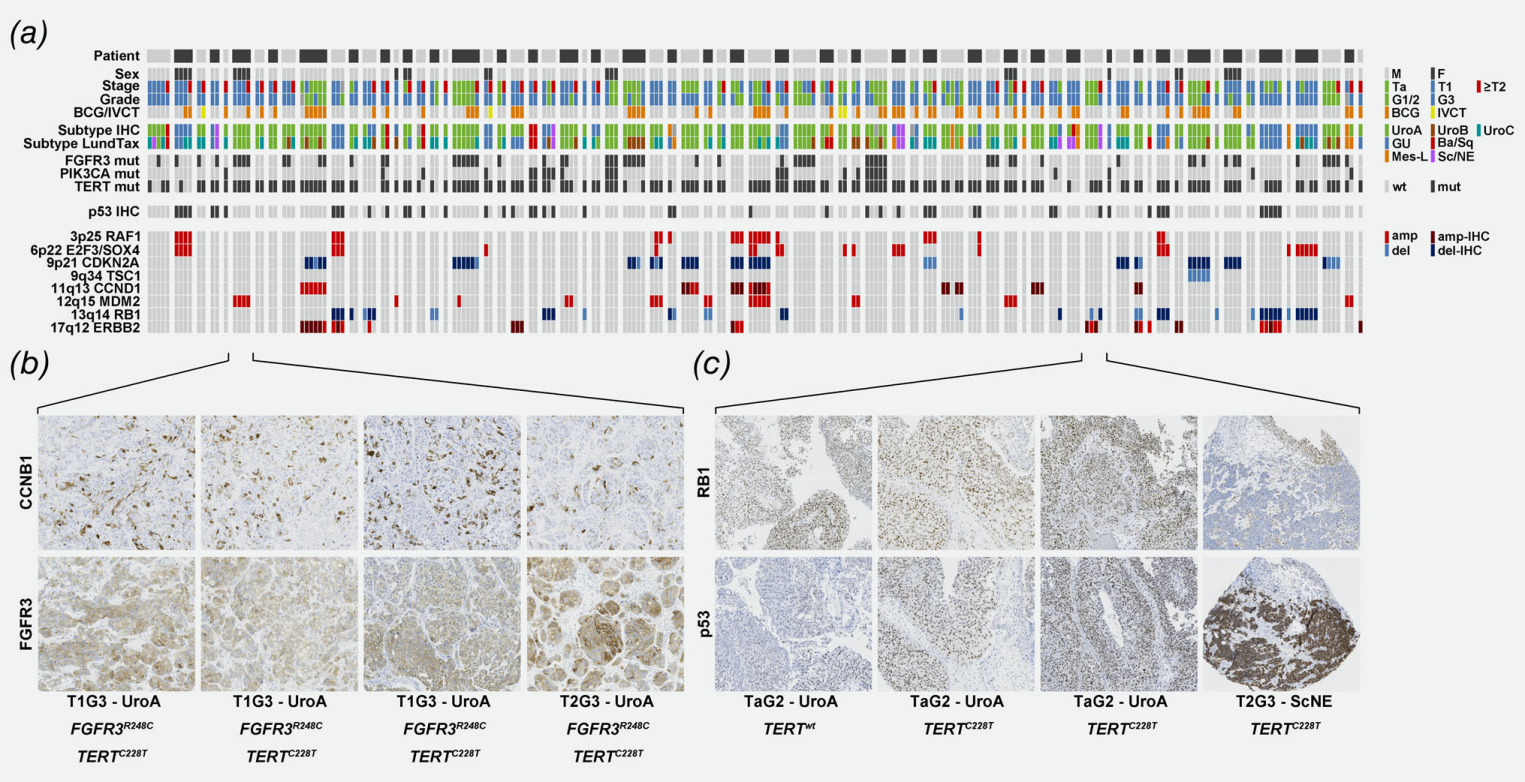
Progression tumors are either identical to previous recurrences or undergo subtype shifts. A summary of clinicopathological data, molecular subtype, mutations, p53‐status and inferred copy number data is shown in (a) ordered by patient, then by tumor number. For inferred copy number alterations, blue boxes indicate gene expression profiles consistent with loss, and red boxes indicate profile consistent with amplification. For CDKN2A, CCND1, RB1 and ERBB2, tumors with IHC‐validated inferred copy number status is indicated with dark blue/dark red boxes. (*b*) Three NMI tumors and the progression tumor from patient 6 show identical molecular profile, including FGFR3 overexpression, *FGFR3* mutation, *TERT* mutation and inferred *MDM2* amplification. (*c*) Three NMI tumors from patient 57 show radically shifted profiles compared to the progression tumor. The NMI tumors are all TaG2 UroA tumors with ERBB2 overexpression, and *TERT* mutation detected in the recurrences but not the primary. The progression tumor is T2G3 of Sc/NE subtype with identical *TERT* mutation, but with molecular changes including RB1 loss, altered p53‐pattern and absence of ERBB2 overexpression. [Color figure can be viewed at http://wileyonlinelibrary.com]

To investigate changes in gene expression during progression, we first focused on genes that are located in genomic regions that harbor frequent copy number alterations. Several such regions including 3p25 (*RAF1*), 6p22 (*E2F3*/*SOX4*), 9p21 (*CDKN2A*), 11q13 (*CCND1*), 12q15 (*MDM2*), 13q14 (*RB1*) and 17q13 (*ERBB2*) were identified, and expression of genes in these regions was investigated. Some patients showed up or downregulation of blocks of genes in defined genomic segments consistent with a copy number alteration event occurring +/−10 genes surrounding the proposed driver gene of each region (Supporting Information Fig. S4). We made putative copy number calls for each patient indicating one or more tumors with gene expression profiles suggesting amplification or deletion at one of the investigated loci (Supporting Information Methods). The data for the regions where the driver gene is known is summarized in Figure 5 *a*. For four inferred copy number alterations, we could investigate expression also at the protein level. Protein levels were altered in the same direction as the predicted copy number alteration for 100/148 cases (*CDKN2A* 39/50, *CCND1* 17/27, *RB1* 27/41 and *ERBB2* 17/30), supporting this approach (Fig. 5 *a*). Most patients showed stable inferred copy number alterations in every analyzed tumor, but for 19 patients at least one alteration differed. By integrating this data with the longitudinal subtyping and mutation data, two patterns of progression emerge: (*i*) Some patients show no molecular difference between the progression tumor and the earlier recurrences. This is exemplified by the patient shown in Figure 5 *b*, with four phenotypically identical UroA tumors that all show *FGFR3* and *TERT* mutations as well as inferred *MDM2* amplification. Proliferation, measured by Cyclin B1 labeling index, was unchanged in the muscle‐invasive progression tumor. (*ii*) Another group of patients showed a radical shift from a Uro‐profile to a more aggressive molecular subtype in the progression tumor, or just prior to it. This is exemplified by the patient in Figure 5 *c*, showing a shift from papillary, UroA tumors to a muscle‐invasive, Neuroendocrine‐like, progression tumor with loss of RB1 and altered p53‐pattern. The same *TERT* mutation present in tumors 2–3 remained present in the progression tumor, but the inferred *ERBB2* amplification status changed.

### Differential gene expression in early *vs*. late tumors is limited to molecular subtype shifts

**Figure 6:**
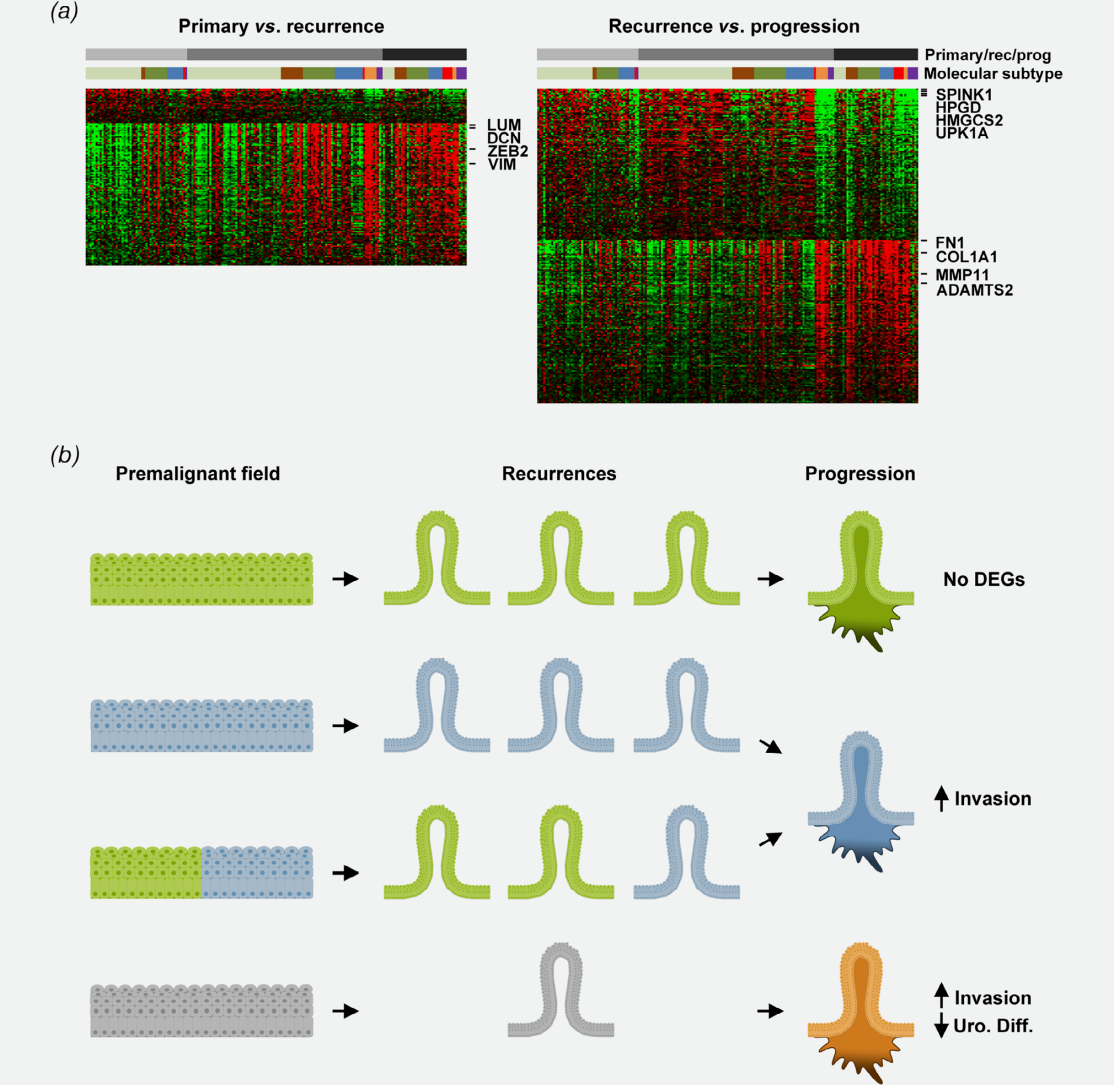
Differential gene expression between primary tumors, recurrences and progression tumors suggests stochastic or punctuated rather than gradual modes of progression. Heatmap in (*a*) (left) showing expression in the full data set of 135 genes differentially expressed between primary tumors and subsequent recurrences. Genes upregulated in recurrences formed a coherent signature and were mainly coding for ECM components (*LUM*, *DCN*) and markers of nonepithelial cells (*ZEB2*, VIM). Heatmap in (*a*) (right) showing expression in the full data set of 239 genes differentially expressed between the last NMI recurrences and subsequent progression tumors. Genes upregulated in progression tumors formed a coherent signature and were mainly coding for ECM components (*FN1*, *COL1A1*) and genes involved in ECM remodeling and invasion (*MMP11, ADAMTS2*). The top downregulated genes in progression tumors were members of a urothelial differentiation signature expressed in luminal‐like subtypes (*SPINK1*, *HPGD*, *HMGCS2* and *UPK1A*). Ordering by molecular subtype, revealed such downregulation primarily in nonluminal‐like tumors and the same analysis, limited to patients with stable Urothelial‐like subtype (UroA, light green, UroB, brown or UroC, dark green) resulted in no significant DEGs. (*b*) Schematic model suggesting different modes of progression in bladder cancer: In the stable modality (first and second panels), patients recur and progress stably within the Uro subtype (green color) or the GU subtype (blue color), suggesting a common origin of all recurrences in a premalignant field that only produces tumors of one subtype. In the unstable modality (third and fourth panels), patients either develop both Uro or GU recurrences and progress with a GU subtype, or progress to a nonluminal‐like subtype (orange color, bottom). Patients that progress with a nonluminal‐like subtype had few prior recurrences that could be of any subtype (gray). The unstable modality of progression involves radical shifts in molecular subtype over the disease course suggesting an underlying premalignant field that is either heterogeneous or evolving to gives rise to various subtypes. [Color figure can be viewed at http://wileyonlinelibrary.com]

To investigate systematic changes in gene expression over the disease course, we performed pairwise comparisons between primary tumors (*n* = 42) and matched subsequent recurrences, and between progression tumors (*n* = 33) and matched previous recurrences. The analyses identified 135 and 239 differentially expressed genes (DEGs; *q* < 0.01), respectively (Supporting Information Table S7). Of 135 DEGs between primary tumors and recurrences, 109 were upregulated in recurrences. These genes were mainly coding for extracellular matrix (ECM) components and markers of mesenchymal cells (Fig. 6 *a*). A likely explanation for this is that recurrences, when sampled, contain a higher proportion of stroma. The 28 DEGs that were downregulated upon first recurrence did not demonstrate any obvious unifying biological theme. Comparison of progression tumors to the last prior recurrence, identified 123 upregulated and 116 downregulated DEGs. Once again, the upregulated genes were mainly coding for ECM components (e.g., *FN1*, *COL1A1*), but also ECM remodeling enzymes (e.g., *MMP11*, *ADAMTS2*) or blood vessel markers (e.g., *SULF1*, *VCAN*), suggesting that either sample composition or the more invasive nature of progression tumors, may underlie these differences. Finally, the top DEGs downregulated in progression tumors included multiple members (*SPINK1*, *HPGD*, *UPK1A* and *HMGCS2*) of the core urothelial differentiation signature that distinguish luminal‐like subtypes (Uro and GU) from their nonluminal counterparts (Ba/Sq, MEs‐like or Sc/NE). Thus, we observed two strong differences in the gene expression profile of progression tumors compared to prior recurrences: an upregulation of ECM‐ or blood vessel‐related genes, and a downregulation of urothelial differentiation signature genes. To determine if the DEGs are driven by a gradual increase in aggressiveness or by a fraction of progression tumors abruptly shifting over to nonluminal‐like subtypes, we limited the analyses to include only patients with stable Uro tumors in Figure 3 *a*. This resulted in no significant DEGs for either of the two pairwise analyses. Thus, progressed tumors without a radical subtype shift did not show any systematic changes in gene expression, and the global differences in the initial analysis of progression tumors were driven by the patients that changed molecular subtype upon progression.

## Discussion

Nonmuscle invasive bladder cancer (NMIBC) is a unique disease in that multiple tumors can be diagnosed and removed allowing longitudinal sampling from the same patient. We did not have data on tumor location within the bladder, but since NMIBCs usually recur in different locations,^13^ we assumed that each resection was complete and each tumor was independent in this sense. Since our aim was to study patients longitudinally, data on multifocality was not collected and all analyses were done on one selected TUR‐BT block. While the prognosis of NMIBC is comparatively good, it is not considered a benign condition, since a proportion of patients progress to muscle‐invasive or metastatic disease despite intravesical treatment with BCG.^36^

Several observations prompted us to conduct our study. First, it is not known to what extent the premalignant field that gives rise to recurrent NMIBC is genetically heterogeneous or oligo‐clonal. Such a genetic heterogeneity has been found in the premalignant lesions in Barrett's esophagus,^5^ and has been linked to progression of breast tumors.^37^ The most detailed genetic studies of the premalignant urothelium has been done in research groups of Dyrskjøt,^8^, ^9^ and Czerniak.^6^, ^7^ Both groups have used multiregion sampling from whole organs to identify tumor‐associated mutations in the noncancerous epithelium. Thomsen *et al*. identify such mutations at low allele‐frequency suggesting spatial intermixing of the premalignant field(s) with wild‐type urothelium.^9^ Several studies have also focused on multifocal or recurrent NMIBC and somatic events, for example, loss of heterozygosity (LOH) on chromosome 9 or *FGFR3* mutation. Hartmann and colleagues showed that early multifocal tumors are either identical, or different but compatible with clonal origin.^38^ Van Tilborg and colleagues constructed hierarchical relationships between NMIBC recurrences using LOH patterns. Their analysis suggested that recurrences are clonal but show a genetic timeline of tumors that did not match their temporal order.^11^ Kompier and colleagues demonstrated that *FGFR3* mutations occur with relative stability in some patients but not in others.^18^ Lindgren and colleagues used several methods to describe remarkably similar molecular profiles across recurrences.^39^ In general, our observations were consistent with these studies, with minor differences which could be explained by our selection of patients with progressive disease. For example, the larger proportion of *FGFR3* wild type tumors in our study can be explained by such selection bias, since *FGFR3* mutation is associated with improved progression‐free survival in NMIBC.^40^, ^41^ The frequency of *FGFR3*, *PIK3CA*, and *TERT* mutations was comparable to previous studies on NMIBC^42^, ^43^ albeit with slight differences attributable to patient selection. Since we studied only progressive disease, it also seems logical that the variability between patients in, for example, mutation instability is larger than in previous recurrence studies. Still, some patients did show the extreme stability previously reported for recurrences, but others had seemingly independent events in each tumor. A limitation regarding the observed mutation patterns is that they were based only on three genes, and were partly based on negative results (wild type), which depend on the assay sensitivity. Furthermore, to test whether within patient stability correlates with genetically clonal/stable *vs*. genetically oligo‐clonal/unstable premalignant fields would require sampling multiple recurrences and several biopsies from normal‐appearing urothelium at each time point.

A strength of our study compared to similar previous studies is that we have access to an additional layer of phenotypic data in molecular subtypes. The vast majority (IHC: 92% and mRNA: 87%) of this cohort was classified as “luminal‐like” subtypes Uro or GU, which in more advanced stages make up only about 50%.^28^, ^30^, ^44^ The dynamics of molecular subtypes in the cohort was interesting: Patients that progressed with the Uro subtype tended to have only prior Uro tumors; patients that progressed with the GU subtype, tended to have either Uro or GU prior tumors; and patient that progressed with nonluminal subtypes had few prior tumors that could be of any subtype. These data explain to some degree why certain subtypes are more frequent at different disease stages. We studied closely the patients that changed molecular subtype, and we could show that true changes in molecular subtype are relatively rare and do not coincide with changes in driver mutations. Instead, subtype change may coincide to some degree with BCG treatment. This hypothesis‐generating finding remains unresolved since our data were hampered by missing data from tumors just prior to, or after BCG‐treatment, which limited the power of the analysis. Furthermore, our analysis of co‐occurrence between subtype shifts and BCG made use of all tumors, even those that clinically might not have been candidates for BCG, further limiting the conclusions of this analysis. The positive co‐occurrence of subtype change and p53‐alteration in recurrences was more robust. This suggests that for patients who progress from a Uro subtype (with low *TP53* mutation frequency) to a more aggressive subtype, the “progressed” invasive tumors have similar *TP53* mutation frequency as *de novo* muscle‐invasive cases among those subtypes. Note that we define a subtype change as the consecutive observation of two different molecular subtypes within one patient. It is not possible to read out from these data whether the molecular subtype of one lesion has changed, or whether the two subtypes have evolved in parallel.

Our analysis of gene expression in regions of copy‐number alterations showed that the rough inference of genetic events from gene expression data was largely confirmed at the protein level. Inferred alterations seemed more stable within patients than driver mutations. Previous studies have indicated that homozygous deletions of *CDKN2A*, a hallmark of advanced Uro tumors, correlate with progression and occur late in development.^45^ We did not observe this pattern, possibly due to difficulty to discriminate homozygous loss from single‐copy loss of chromosome 9, a universal event also in very early bladder tumors.^42^, ^46^ To definitively test the accuracy of inferring copy number alterations would require copy‐number data from a similar series of recurrences, which was not possible due to the quality and amount of DNA obtained. If the validity of this approach can be established, copy number alterations inferred by patterns of RNA abundance may be an efficient way to study similarity between NMIBC recurrences and premalignant urothelium.

The early division between Uro and GU subtypes often persisted over multiple recurrences, which is in line with the mutually exclusive nature of these subtypes in a recent study on UBC and concomitant histologic variants.^47^ The two entities Uro and GU, identified by unsupervised clustering of RNA profiles, mirror the marker profiles of hyperplasia and carcinoma *in situ*, respectively.^48^ The molecular subtypes, therefore, provide a phenotypic correlate to the well‐established “two pathway model” of bladder tumorigenesis that is rooted in histology and genetics.^39^, ^49^, ^50^

Taken together, our data is fully compatible with a model in which a clonal premalignant field is the precursor of semi‐independent recurrences. However, recent work has demonstrated the similarity between upper urinary tract urothelial cancer and bladder recurrences suggesting that recurrence due to dissemination may occur in this particular context.^51^ By introducing the concept of molecular subtypes, we contextualize also the well‐established two‐pathway model for luminal‐like tumors. Finally, we add to the model two fundamentally different modes of progression: For most patients, progression occurs without any change in molecular subtype. Most often, the premalignant field only gives rise to Uro tumors (Fig. 6 *b*, top). These progression tumors have no DEGs, or other systematic molecular changes compared to earlier recurrences. Less frequently, patients may also recur with GU tumor only (Fig. 6 *b*, second panel). In its other modality, progression is coupled to an abrupt subtype change suggesting that field heterogeneity, possibly caused by genomic instability within the field, could produce tumors of different molecular subtypes. For Uro to GU transitions (Fig. 6 *b*, third panel) this manifests in upregulated invasion related genes, and for patients transitioning to nonluminal subtypes (Fig. 6 *b*, bottom) it also involves downregulation of urothelial differentiation genes. Both the two last scenarios involve subtype change and may also coincide with acquiring abnormal p53 staining pattern indicative of *TP53* mutation. We note that our suggested “punctuated” progression model is incompatible with the concept of gradual increases in aggressiveness culminating in invasion and clinical progression. Instead, we suggest that radical changes, when observed in overt tumors, are manifestations of a “sea change”, or instability, in the premalignant precursor field enabling it to (also) produce tumors of a new type.

The majority of patients, however, progress with the first type, that is, stable Uro disease. These patients will likely be difficult to identify at baseline from patients with Uro recurrences that never progress. The difference may even be stochastic, that is, stable Uro patients that progress may be impossible to separate from those that do not. Another possibility is that factors already present at tumor initiation specify recurrence and progression risk individually for each patient. Since all tumors from stable Uro patients are similar, any tumor may serve as a representative index tumor for that patient. The second group of patients has opposite behavior with drastic changes in molecular profiles before or during progression. A robust change in molecular subtype or an occurrence of NMIBC with nonluminal subtype would indicate a patient may be of this type. For such patients, risk‐prediction based on biomarkers from a single index tumor will be inadequate since they represent only one of many molecular manifestations possible in the patient's bladder.

## Supporting information


**Appendix S1.** Supporting Information.

**Figure S1** Quantitative analysis of molecular subtype classification scores within patients. Each data point is one tumor colored in (*a*) by IHC‐based molecular subtype, and in (*b*) by mRNA‐based molecular subtype. Y‐axis values indicate each tumors correlation in subtype score to the other tumors of the same patient. Patients are ordered from left to right into two patient categories separated by a dashed line; Patients with one or more tumors showing low correlation, and patients with all tumors showing stable high correlation. Only tumors in the first patient category are considered to have truly undergone a change in molecular subtype.

**Figure S2** Mutation frequency stratified by primary, recurrent and progressive tumor status, and by molecular subtype. Stacked barplots in (*a*) show no significant difference in frequency of *FGFR3*, *PIK3CA* and *TERT* mutation between primary, recurrent and progressive tumors. Barplots in (*b*) show significant enrichment of *FGFR3* mutations in Uro tumors, significant depletion of *FGFR3* mutations in GU tumors and significant enrichment of *PIK3CA* mutations in Ba/Sq tumors, despite a low number of cases in this subtype. Barplots in (c) show significant enrichment of p53‐altered pattern in GU tumors and depletion in Uro tumors.

**Figure S3** Patient number 33 demonstrated change in molecular subtype and p53‐alterations after long term remission after BCG treatment. The top panel shows the timeline for patient number 33, indicating stage, grade and molecular subtype of the tumors. Mutation data were available and is indicated for four tumors. BCG treatment followed by a remission of more than 10 years occurred after tumor number four. The first relapse after long‐term remission was also an Uro tumor but with a *FGFR3* mutation not present in tumors 1–4. The patient then experienced a subtype shift such that tumors number 6 and 8 were classified as GU. IHC from tumor number 7 was missing. Altered p53‐staining (overexpression) coincided with shift from Uro to GU subtypes.

**Figure S4** Co‐ordinated gene expression across recurrences at loci harboring frequent copy number alterations. The top panel summarizes data ordered first by patient, then by tumor number. Alternating dark and light gray bars indicate different patients. Dark gray boxes indicate female patients, and the presence of gene mutations. For stage and grade, green boxes indicate Ta/G1‐G2, blue boxes indicate T1/G3, and red boxes indicate stage ≥T2. Orange boxes indicate BCG treated tumors, and yellow boxes indicate tumors treated with intravesical chemotherapy (IVCT). Molecular subtype classification is color‐coded as in Figure 3. Heatmaps show median centered gene expression at loci with frequent copy number changes in bladder cancer. Yellow indicates high expression and blue indicates low expression. Each panel shows the genes of one locus in genomic order, and the target gene is indicated (blue = tumor suppressor, red = oncogene). Boxes below each panel indicate cases with gene expression profile consistent with genomic alteration.

**Table S1** Supporting InfoItem

**Table S2** Supporting InfoItem

**Table S3** Supporting InfoItem

**Table S4** Supporting InfoItem

**Table S5** Supporting InfoItem

**Table S6** Supporting InfoItem

**Table S7** Supporting InfoItem

## Data Availability

Raw and processed gene expression data is available through Gene Expression Omnibus under the accession number GSE128959. All data of other types is available in Supporting Information Table S1.
