# Supplementary material for: Molecular changes during progression from nonmuscle invasive to advanced urothelial carcinoma
Source: Int J Cancer. 2019 Nov 14;146(9):2636–47. doi: 10.1002/ijc.32737 (PMC7079000; doi:10.1002/ijc.32737)
Supplement: Supplementary file 1 — Appendix S1. Supporting Information. [file IJC-146-2636-s001.doc]

**Supplementary Methods**

**Inferred copy number analysis**

Gene expression data was ordered by chromosomal location (genes) and by temporal order within patients (samples). Expression levels for known targets/target regions of genomic amplifications or deletions, plus minus ten genes surrounding the (putative) target gene were extracted. Targets/regions were identified from the literature and included Chr 1q21 (MCL1) (Eriksson et al. 2013), 3p25 (PPARG/RAF1) (Simon et al. 2001), 6p22 (E2F3/SOX4) (Eriksson et al. 2013, Knowles and Hurst 2015), 8q22 (YWHAZ) (Yu et al. 2019), 8p11 (FGFR1) (Knowles and Hurst 2015), 9p21 (CDKN2A) (Knowles and Hurst 2015), 9q34 (TSC1) (Knowles and Hurst 2015), 11q13 (CCND1) (Knowles and Hurst 2015), 12q15 (MDM2) (Knowles and Hurst 2015), 13q14 (RB1) (Knowles and Hurst 2015), 17q12 (ERBB2) (Eriksson et al. 2017, Knowles and Hurst 2015). Multiple gene expression profiles from each patient were then investigated for block patterns of up- or downregulation consistent with copy number alteration. Inferred alterations were called manually based on evaluating the gene expression data with regard to: a) Relative expression of the putative target gene, b) coherent up- or downregulation of surrounding genes in the region, c) coherent expression profile of the region between samples from the same patient. Called cases are indicated in Supplementary Figure S4. along with a heatmap of gene expression in the relevant surrounding genes. For four genes, single IHC-markers were compared to gene expression data. Protein expression ≤ 0.5 (range 0-3) was considered low, consistent with copy number loss, and protein expression ≥ 1.5 was considered high.

References

Eriksson P, Aine M, Sjödahl G, Staaf J, Lindgren D, Höglund M. Detailed Analysis of Focal Chromosome Arm 1q and 6p Amplifications in Urothelial Carcinoma Reveals Complex Genomic Events on 1q, and SOX4 as a Possible Auxiliary Target on 6p. PLoS One. 2013 Jun 18;8(6):e67222.

Simon R, Richter J, Wagner U, Fijan A, Bruderer J, Schmid U, Ackermann D, Maurer R, Alund G, Knönagel H, Rist M, Wilber K, Anabitarte M, Hering F, Hardmeier T, Schönenberger A, Flury R, Jäger P, Fehr JL, Schraml P, Moch H, Mihatsch MJ, Gasser T, Sauter G. High-throughput tissue microarray analysis of 3p25 (RAF1) and 8p12 (FGFR1) copy number alterations in urinary bladder cancer. Cancer Res. 2001 Jun 1;61(11):4514-9.

Knowles MA, Hurst CD. Molecular biology of bladder cancer: new insights into pathogenesis and clinical diversity. Nat Rev Cancer. 2015 Jan;15(1):25-41.

Yu CC, Li CF, Chen IH, Lai MT, Lin ZJ, Korla PK, Chai CY, Ko G, Chen CM, Hwang T, Lee SC, Sheu JJ. YWHAZ amplification/overexpression defines aggressive bladder cancer and contributes to chemo-/radio-resistance by suppressing caspase-mediated apoptosis. J Pathol. 2019 Aug;248(4):476-487.

Eriksson P, Sjödahl G, Chebil G, Liedberg F, Höglund M. HER2 and EGFR amplification and expression in urothelial carcinoma occurs in distinct biological and molecular contexts. Oncotarget. 2017 Jul 25;8(30):48905-48914.
