# Supplementary figures and images for: Molecular changes during progression from nonmuscle invasive to advanced urothelial carcinoma
Source: Int J Cancer. 2019 Nov 14;146(9):2636–47. doi: 10.1002/ijc.32737 (PMC7079000; doi:10.1002/ijc.32737)

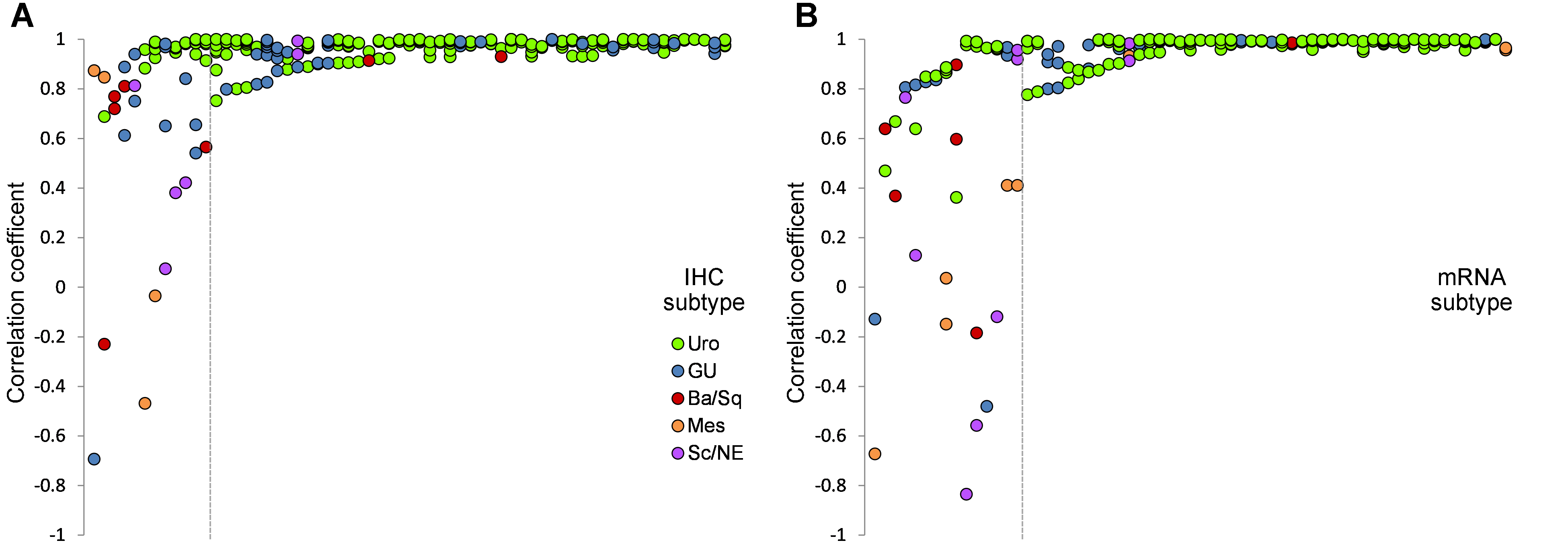

Supplement: Supplementary file 2 — Figure S1 Quantitative analysis of molecular subtype classification scores within patients. Each data point is one tumor colored in (a) by IHC‐based molecular subtype, and in (b) by mRNA‐based molecular subtype. Y‐axis values indicate each tumors correlation in subtype score to the other tumors of the same patient. Patients are ordered from left to right into two patient categories separated by a dashed line; Patients with one or more tumors showing low correlation, and patients with all tumors showing stable high correlation. Only tumors in the first patient category are considered to have truly undergone a change in molecular subtype. [file IJC-146-2636-s002.tif]

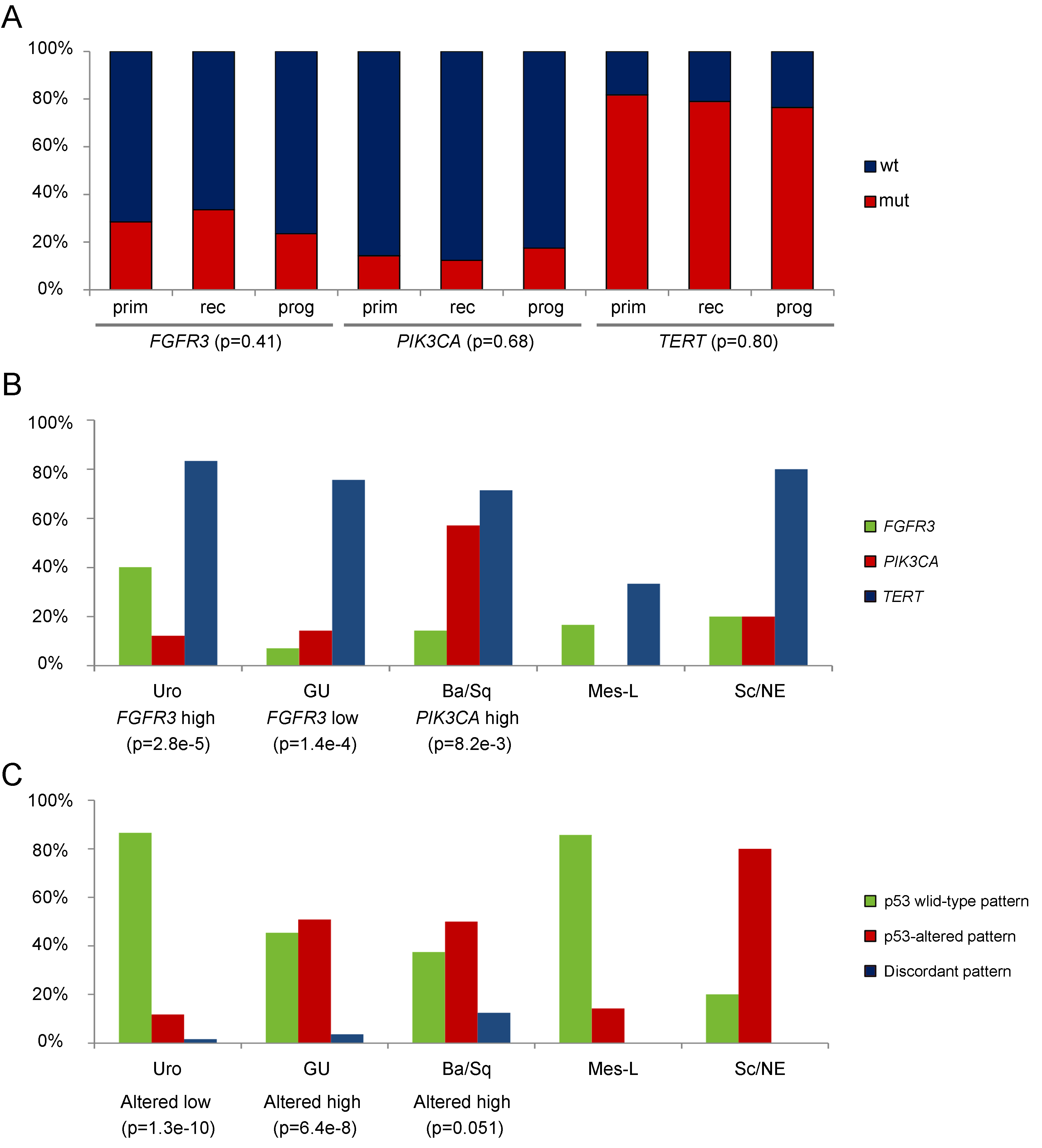

Supplement: Supplementary file 3 — Figure S2 Mutation frequency stratified by primary, recurrent and progressive tumor status, and by molecular subtype. Stacked barplots in (a) show no significant difference in frequency of FGFR3, PIK3CA and TERT mutation between primary, recurrent and progressive tumors. Barplots in (b) show significant enrichment of FGFR3 mutations in Uro tumors, significant depletion of FGFR3 mutations in GU tumors and significant enrichment of PIK3CA mutations in Ba/Sq tumors, despite a low number of cases in this subtype. Barplots in (c) show significant enrichment of p53‐altered pattern in GU tumors and depletion in Uro tumors. [file IJC-146-2636-s003.tif]

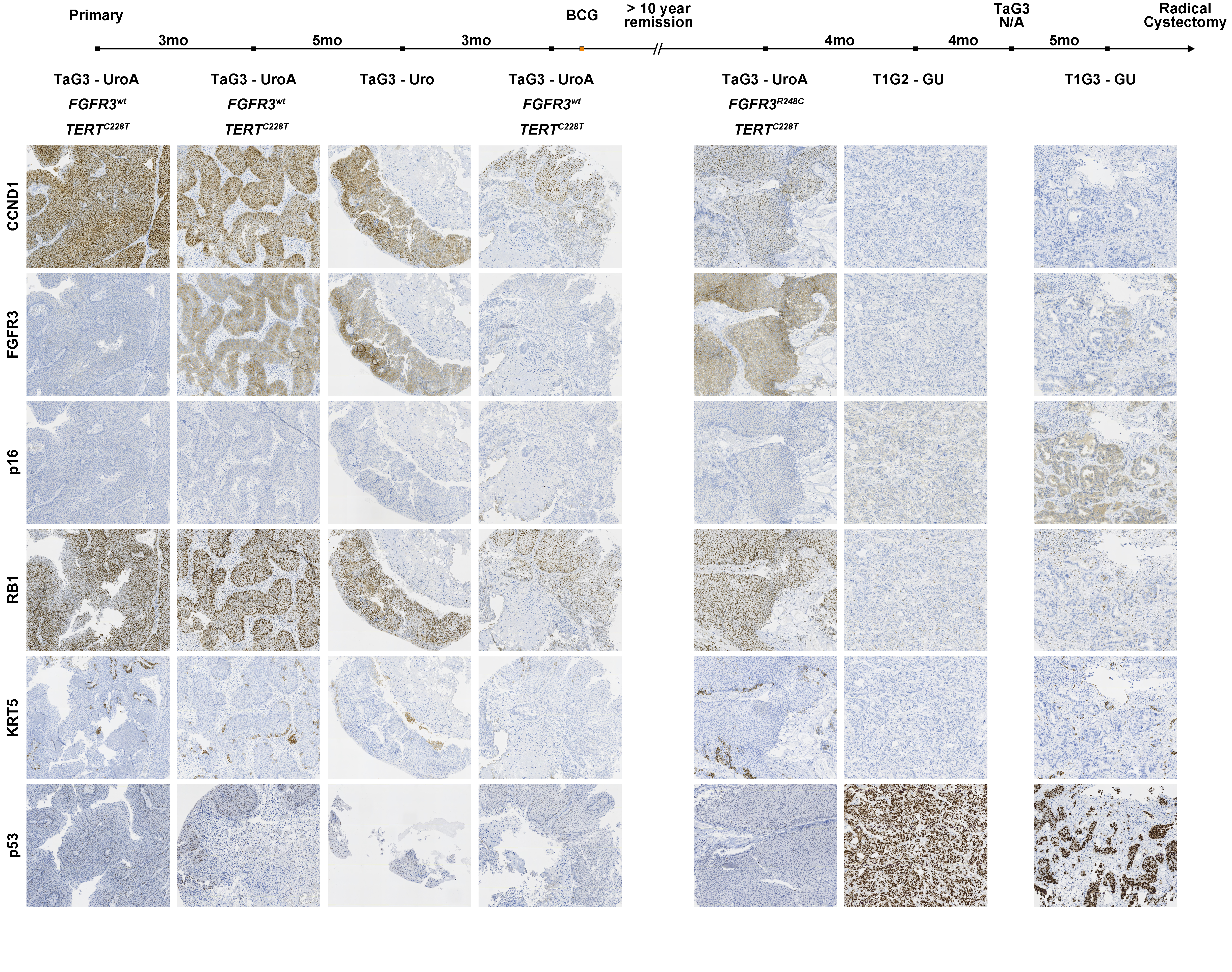

Supplement: Supplementary file 4 — Figure S3 Patient number 33 demonstrated change in molecular subtype and p53‐alterations after long term remission after BCG treatment. The top panel shows the timeline for patient number 33, indicating stage, grade and molecular subtype of the tumors. Mutation data were available and is indicated for four tumors. BCG treatment followed by a remission of more than 10 years occurred after tumor number four. The first relapse after long‐term remission was also an Uro tumor but with a FGFR3 mutation not present in tumors 1–4. The patient then experienced a subtype shift such that tumors number 6 and 8 were classified as GU. IHC from tumor number 7 was missing. Altered p53‐staining (overexpression) coincided with shift from Uro to GU subtypes. [file IJC-146-2636-s004.tif]

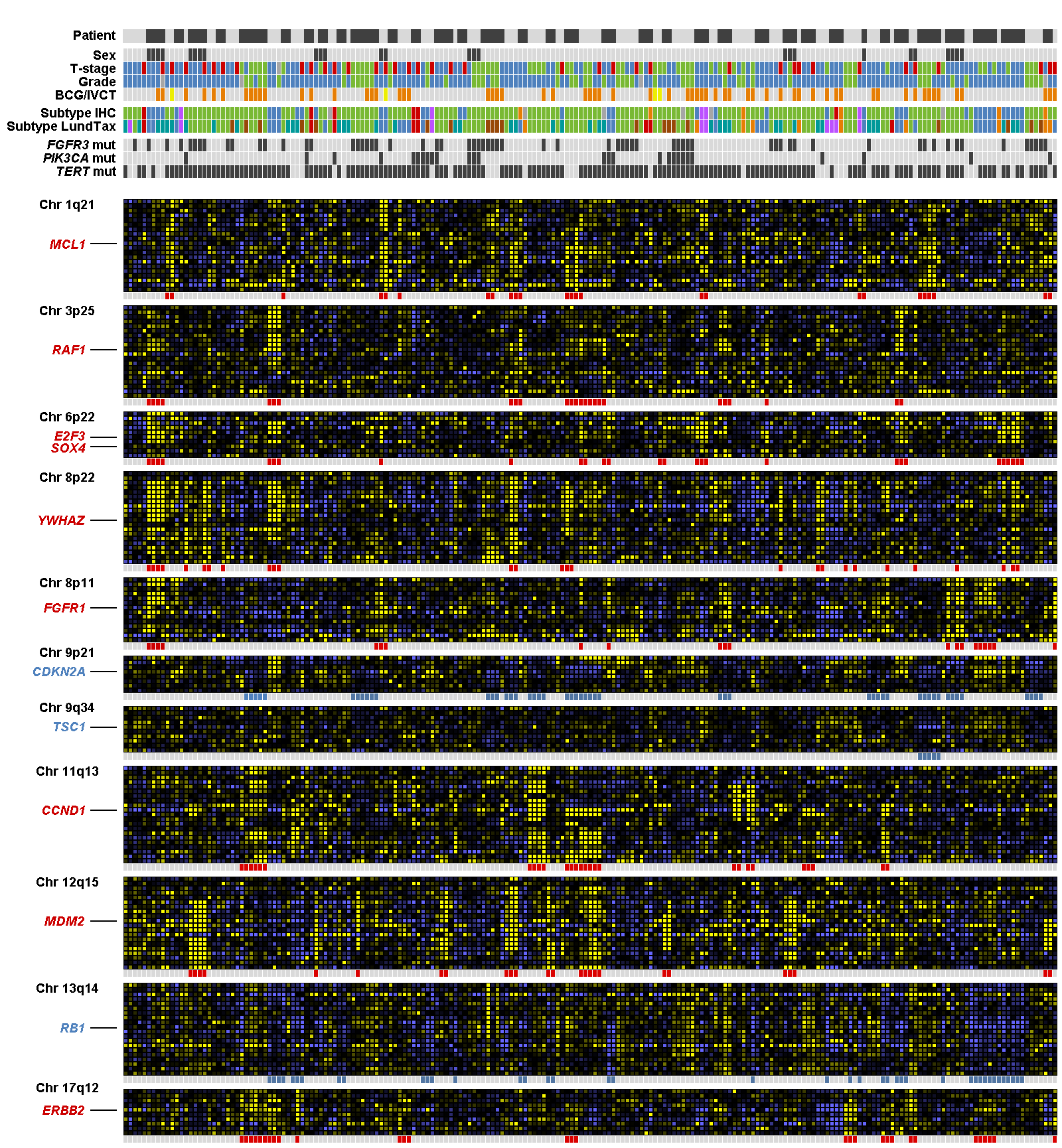

Supplement: Supplementary file 5 — Figure S4 Co‐ordinated gene expression across recurrences at loci harboring frequent copy number alterations. The top panel summarizes data ordered first by patient, then by tumor number. Alternating dark and light gray bars indicate different patients. Dark gray boxes indicate female patients, and the presence of gene mutations. For stage and grade, green boxes indicate Ta/G1‐G2, blue boxes indicate T1/G3, and red boxes indicate stage ≥T2. Orange boxes indicate BCG treated tumors, and yellow boxes indicate tumors treated with intravesical chemotherapy (IVCT). Molecular subtype classification is color‐coded as in Figure 3. Heatmaps show median centered gene expression at loci with frequent copy number changes in bladder cancer. Yellow indicates high expression and blue indicates low expression. Each panel shows the genes of one locus in genomic order, and the target gene is indicated (blue = tumor suppressor, red = oncogene). Boxes below each panel indicate cases with gene expression profile consistent with genomic alteration. [file IJC-146-2636-s005.tif]
