# Supplementary material for: Molecular changes during progression from nonmuscle invasive to advanced urothelial carcinoma
Source: Int J Cancer. 2019 Nov 14;146(9):2636–47. doi: 10.1002/ijc.32737 (PMC7079000; doi:10.1002/ijc.32737)
Supplement: Supplementary file 7 — Table S2 Supporting InfoItem [file IJC-146-2636-s007.pdf]

| Marker | Catalog No. | Vendor | Dilution | Evaluation |
|---|---|---|---|---|
| CCNB1 | 1495-1 | Epitomics | 1:100 | Perc. |
| CCND1 | M3635 | Dako (Agilent) | 1:100 | Perc./Intensity |
| CDH1 | M3612 | Dako (Agilent) | 1:200 | Intensity |
| EPCAM | M3525 | Dako (Agilent) | 1:40 | Intensity |
| ERBB2 | 790-2991 | Ventana | RTU | Intensity |
| FGFR3 | #4574 | Cell Signaling | 1:40 | Intensity |
| FOXA1 | ab40868 | Abcam | 1:200 | Intensity |
| GATA3 | #5852 | Cell Signaling | 1:800 | Perc./Intensity |
| KRT14 | MS-115 | Lab Vision | 1:200 | Perc./Intensity |
| KRT5 | RM-2106 | Lab Vision | 1:200 | Perc./Intensity |
| CDKN2A (p16) | #550834 | BD Biosciences | 1:50 | Intensity |
| RB1 | #9309 | Cell Signaling | 1:100 | Perc. |
| p53 | M7001 | Dako (Agilent) | 1:100 | Pattern |
| TUBB2B | LS-B4190-50 | LifeSpan | 1:200 | Intensity |
| VIM | M0725 | Dako (Agilent) | 1:200 | Perc./Intensity |
| ZEB2 | 61095 | Active Motif | 1:500 | Intensity |
