# Supplementary material for: Molecular changes during progression from nonmuscle invasive to advanced urothelial carcinoma
Source: Int J Cancer. 2019 Nov 14;146(9):2636–47. doi: 10.1002/ijc.32737 (PMC7079000; doi:10.1002/ijc.32737)
Supplement: Supplementary file 8 — Table S3 Supporting InfoItem [file IJC-146-2636-s008.pdf]

DNA extraction yield versus # detected mutations

Quintile	n	DNA µg	FGFR3mut	PIK3CAmut	TERTmut	Total mut
1	40	7.1-17.1	20	6	34	60
2	40	4.4-7.1	14	5	35	54
3	41	2.4-4.4	9	6	36	51
4	41	1.1-2.4	12	6	37	55
5	41	0.1-1.1	11	6	30	47

Tumor ESTIMATE score versus # detected mutations

Quintile	n	ESTIMATE	FGFR3mut	PIK3CAmut	TERTmut	Total mut
1	39	93-98%	15	3	35	53
2	40	88-93%	17	6	28	51
3	40	83-88%	21	9	37	67
4	40	70-82%	7	6	39	52
5	40	41-70%	7	3	31	41

Tumor ESTIMATE score versus molecular subtype (mRNA)

Quintile	n	ESTIMATE	Uro	GU	Ba/Sq	Mes-like	Sc/NE
1	39	93-98%	34	3	0	0	2
2	40	88-93%	29	8	0	0	3
3	40	83-88%	34	4	1	0	1
4	40	70-82%	24	11	2	1	2
5	40	41-70%	20	5	5	9	1
