# Supplementary material for: Molecular changes during progression from nonmuscle invasive to advanced urothelial carcinoma
Source: Int J Cancer. 2019 Nov 14;146(9):2636–47. doi: 10.1002/ijc.32737 (PMC7079000; doi:10.1002/ijc.32737)
Supplement: Supplementary file 9 — Table S4 Supporting InfoItem [file IJC-146-2636-s009.pdf]

| Gene | Mutation | N, tumors analyzed | N, tumors | N, patients (of 71 analyzed) |
|---|---|---|---|---|
| FGFR3 | R248C | 219 | 9 | 6 |
| | S249C | 219 | 29 | 13 |
| | G372C | 219 | 1 | 1 |
| | G372S* | 219 | 1 | 1 |
| | S373C | 219 | 6 | 1 |
| | Y375C | 219 | 13 | 6 |
| | G382R | 219 | 5 | 2 |
| | A393E | 219 | 0 | 0 |
| | K652E** | 213 | 6 | 2 |
| | | | | |
| PIK3CA | E542K | 219 | 8 | 5 |
| | E545G | 219 | 0 | 0 |
| | E545K | 219 | 15 | 8 |
| | H1047R | 219 | 7 | 3 |
| | H1047L*** | 219 | 2 | 2 |
| | | | | |
| TERT | C228T | 215 | 159 | 59 |
| | C228A | 215 | 1 | 1 |
| | C242T | 215 | 8 | 8 |
| | C250T | 215 | 17 | 12 |

* This mutation was detected by the assay designed for the G372C mutation

** Two assays were used for codon 652, but only the K652E mutation was detected

*** These mutations were detected by the assay designed for the H1047R mutation
