# Supplementary material for: Molecular changes during progression from nonmuscle invasive to advanced urothelial carcinoma
Source: Int J Cancer. 2019 Nov 14;146(9):2636–47. doi: 10.1002/ijc.32737 (PMC7079000; doi:10.1002/ijc.32737)
Supplement: Supplementary file 10 — Table S5 Supporting InfoItem [file IJC-146-2636-s010.pdf]

No BCG			BCG coinciding			BCG not coinciding			BCG unknown		
**Subtype change**	**Mut change**	**Prog Type**	**Subtype change**	**Mut change**	**Prog Type**	**Subtype change**	**Mut change**	**Prog Type**	**Subtype change**	**Mut change**	**Prog Type**
Uro → ScNE (RNA)	-C228T	MIBC (pt1)	Uro → Ba/Sq (RNA)	72C -Y375C -H1[illegible]	MIBC (pt14)	GU → Mes (RNA)	-C228T	RC (pt32)	Uro → ScNE (RNA+IHC)	ID	MIBC (pt57)
ScNE → Uro (RNA)	+R248C	MIBC (pt1)	Uro → Ba/Sq (RNA)	+E542K -C228T	MIBC (pt17)	Uro → GU (IHC)	N/A	RC (pt33)			
Uro → Ba/Sq (RNA+IHC)	ID	MIBC (pt1)	Mes → GU (IHC)	ID	RC (pt32)	Uro → Ba/Sq (RNA)	ID	MIBC (pt41)			
GU → ScNE (RNA+IHC)	+S249C	M+ (pt4)	Uro → Mes (IHC)	ID	MIBC (pt41)	Uro → Mes (RNA)	-C228T	MIBC (pt48)			
Uro → GU (RNA)	ID	MIBC (pt16)	Mes → ScNE (RNA)	-C250T	RC (pt45)	Uro → Mes (RNA)	ID	MIBC (pt48)			
GU → Uro (RNA)	N/A	MIBC (pt25)	Uro → GU (RNA)	ID	MIBC (pt46)	ScNE → GU (IHC)	N/A	M+ (pt55)			
[Uro (RNA), GU (IHC)] → ScNE (RNA+IHC)	ID	MIBC (pt25)	Mes → Uro (RNA)	+C228T	MIBC (pt48)	Ba/Sq → Mes (IHC)	ID	MIBC (pt56)			
GU → Ba/Sq (IHC)	ID	MIBC (pt58)	Uro → ScNE (RNA + IHC)	N/A	M+ (pt55)	Uro → Mes (IHC)	N/A	MIBC (pt60)			
			Uro → Ba/Sq (IHC)	-C228T	MIBC (pt56)	Mes → Uro (IHC)	N/A	MIBC(pt60)			
			GU → Mes (RNA)	ID	MIBC (pt63)						
			Uro → GU (RNA)	-C228T	MIBC (pt64)						
