# Supplementary material for: Molecular changes during progression from nonmuscle invasive to advanced urothelial carcinoma
Source: Int J Cancer. 2019 Nov 14;146(9):2636–47. doi: 10.1002/ijc.32737 (PMC7079000; doi:10.1002/ijc.32737)
Supplement: Supplementary file 11 — Table S6 Supporting InfoItem [file IJC-146-2636-s011.pdf]

p53 pattern	Total cases	Concordant	Discordant*	Single core
Wild-type (wt)	221	144	6	70
Overexpression (oe)	57	40	3	14
Complete absence (ca)	9	6	3	0
Cytoplasmic (cy)	1	1	0	0

* 6 discordant tumors, 3 wt/oe, and 3 wt/ca
